# Direct Synthesis of Ultrathin Hexagonal Boron Nitride Films on Si(001)

**DOI:** 10.1021/acs.nanolett.6c01435

**Published:** 2026-06-03

**Authors:** Max Franck, Jaroslaw Dabrowski, Markus Andreas Schubert, Gianfranco Sfuncia, Giuseppe Nicotra, Christian Wenger, Mindaugas Lukosius

**Affiliations:** † IHP − Leibniz Institute for High Performance Microelectronics, Im Technologiepark 25, 15236 Frankfurt (Oder), Germany; ‡ Istituto per la Microelettronica e Microsistemi (CNR-IMM), Ottava strada, 5 (Zona Industriale), 95121 Catania, Italy; § Semiconductor Materials, BTU Cottbus-Senftenberg, Platz der Deutschen Einheit 1, 03046 Cottbus, Germany

**Keywords:** hexagonal boron nitride, 2D materials, chemical vapor deposition, borazine, CMOS compatible

## Abstract

Synthesis of hexagonal boron nitride (hBN) on CMOS-compatible substrates is desirable for large-scale fabrication of hBN-based micro- and optoelectronic devices. However, growth of well-oriented, high-quality hBN films via chemical vapor deposition (CVD) on silicon substrates is still challenging. We report a new method to synthesize ultrathin, well-oriented hBN films on Si(001) substrates, based on a continuous flow CVD process using borazine as a single-source precursor. In addition, spectroscopic ellipsometry is explored as a fast, nondestructive method to determine the thickness, refractive index, and optical bandgap of the grown hBN films. The resulting hBN films exhibit a thickness of 7 ± 1 layers, near perfect stoichiometry, a surface roughness of 1.1 nm, a refractive index of 2.17 at 633 nm, and an optical bandgap of 5.89 eV. The use of borazine precursor avoids the availability of reactive nitrogen species and thus prevents the formation of an amorphous SiN_*x*_ interlayer at the Si interface.

## Graphical abstract

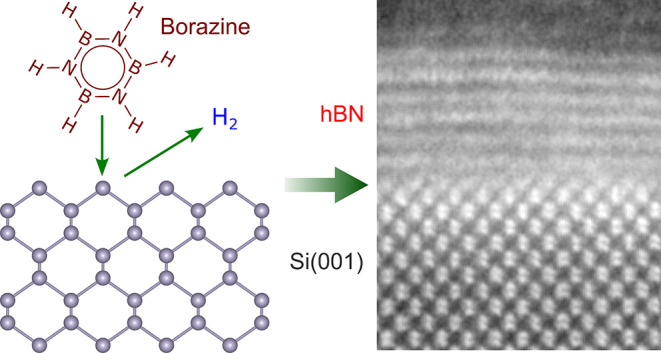

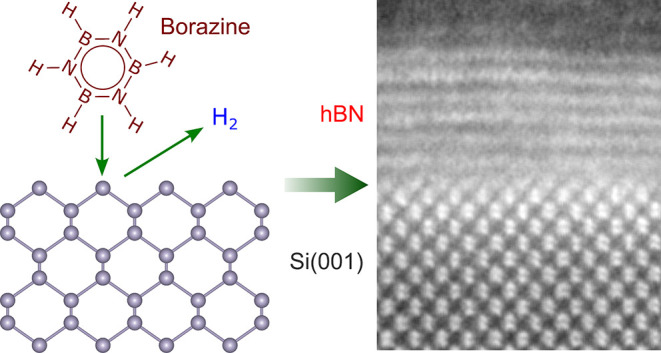

Hexagonal boron nitride (hBN) is a two-dimensional insulator structurally analogous to graphene, with a range of interesting applications including deep ultraviolet optoelectronics,^1,2^ protection layers for high mobility graphene-based (opto-)­electronic devices,^3–6^ and solid-state thermal neutron detectors.^7,8^ By far the most common method of hBN thin film synthesis is chemical vapor deposition (CVD) on catalytic transition metal substrates like Cu and Ni, producing large-area, high-quality mono- and multilayers.^9–13^ To fabricate actual devices, however, transfer of the hBN films is then required, which limits the scalability of the manufacturing process. Additionally, it was shown that the transfer processes leave behind residual metal contaminations at concentrations incompatible with the stringent purity requirements of CMOS manufacturing lines.^14–16^

Direct synthesis of hBN thin films on CMOS-compatible substrates, such as Si and Ge, as well as dielectrics such as SiO_2_, Si_3_N_4_, and sapphire, eliminates the problem of metal contaminations after transfer. Furthermore, depending on the specific application, it could enable deposition of hBN directly as part of the device fabrication process and avoid transfer altogether, which makes it a highly promising approach. Despite these benefits, this area of research has received relatively little attention compared to hBN growth on metal substrates. So far, the research that has been published is predominantly focused on the deposition of hBN on dielectric substrates, especially sapphire, with reports ranging from several hundred nanometer thick, nanocrystalline hBN films^17–19^ to high-quality, well-aligned growth of a few to several dozen hBN layers,^20–26^ employing a wide range of precursors, pretreatments, pressures and process temperatures. Nevertheless, the mechanisms leading to either of these growth modes are not well understood yet and precise control over the number of hBN layers remains challenging. On Ge substrates, synthesis of polycrystalline mono- and few-layers as well as single-crystalline monolayers has been reported.^27–31^ The main disadvantage of Ge is its relatively low melting point of 938 °C, which strongly limits the applicable growth temperatures and leads to roughening of the Ge surface via sublimation at *T* > 900 °C. The sublimation can be suppressed by placing the substrate growth-face-down onto another substrate, but this results in extremely low growth rates (0.1–1 ML/h) and might not be an option for larger-scale CVD processes.

Silicon, on the other hand, has a much higher melting point (1414 °C), allowing for higher growth temperatures while maintaining a flat interface. In addition, as the semiconductor industry’s principal material, it is inexpensive and could facilitate the large-scale synthesis of hBN thin films. Since Si is a noncatalytic substrate for hBN growth, achieving hBN films of high quality and with a controllable number of layers that are well-oriented parallel to the Si interface instead of forming randomly oriented nanocrystallites has proven challenging, and the available literature is extremely limited. Mostly, growth of nanocrystalline hBN films on Si(001) and Si(111) surfaces has been reported, with thicknesses ranging from 400 nm to 2.3 μm.^32,33,18^ As precursors, both single-source (ammonia borane) and separate N and B precursors (e.g., ammonia [NH_3_] + triethylborane [TEB]) have been used and growth temperatures ranged between 1100–1350 °C. Another study investigated the impact of nitridation and carbidization pretreatments of Si(111) substrates on the growth of sp^2^-BN at 1300 °C using NH_3_ and TEB precursors with the addition of a small amount of silane.^34^ No benefit of the pretreatment was found over growth on untreated Si(111), and the resulting films were mostly amorphous with a small fraction of nanocrystalline hBN. Recently, a pulsed flow mode CVD process was proposed, consisting of alternating TEB and NH_3_ pulses followed by a H_2_ purge cycle at 1100 °C, which resulted in the formation of well-oriented few-layer hBN on Si(111) substrates.^35^ Additionally, the formation of a 1.2 nm thick, amorphous SiN_*x*_ interlayer between the Si and hBN was observed, which might be related to reactions between Si and ammonia in the early stages of growth. The impact of such an interlayer on the further usability of the hBN films is currently unknown, as are alternative routes toward layered hBN/Si films. Therefore, further development of hBN growth processes on Si substrates is required.

In this letter, we report a method to synthesize ultrathin, well-oriented, layered hBN films on Si(001) substrates without the formation of an amorphous interlayer. It is based on a conventional continuous flow CVD process using borazine (B_3_N_3_H_6_) as a single-source precursor. In addition to a thorough characterization of the grown films, spectroscopic ellipsometry is explored as a fast and nondestructive method to obtain complementary quality indicators, such as the optical bandgap, directly on the growth substrate. The choice of borazine as a single-source precursor avoids the use of ammonia, which can react with Si to form silicon nitrides. Compared to ammonia borane, another single-source hBN precursor, borazine offers better stability of the precursor feed rate throughout the process and a simpler experimental setup, as it can be delivered to the reactor via a bubbler setup instead of sublimation.

The findings in this letter represent the results of an optimized set of growth conditions at 10^–3^ mbar and 900 °C, based on a comprehensive optimization study conducted for hBN growth on Ge(001) substrates. The temperatures of the sample surface and of the precursor gas prior to entering the reactor chamber, as well as the local borazine partial pressure, were identified as key parameters influencing the quality of the hBN films. The crystallinity increases with increasing growth temperature, but the crystallites are randomly oriented instead of aligned parallel to the Si interface. A very low growth rate of ∼ 2 ML/h, facilitated by a low local borazine partial pressure of approximately 10^–4^ mbar at the sample surface, then yields the desired well-oriented hBN layers. In our setup, the borazine is transported to the reactor chamber via Ar carrier gas, and injected into the reactor via a stainless-steel tube. Sufficient distance between the injector tube and the heated sample ensures a low and uniform borazine partial pressure at the sample surface and also limits the temperature of the precursor gas prior to entering the reactor chamber, which suppresses unwanted gas-phase reactions that might negatively impact the crystallinity of the hBN films. For further information about the experimental setup and the influence of the process parameters refer to our previous publications,^36,31^ and the Supporting Information.

After 240 min of growth under optimized conditions, the deposited hBN films exhibit a thickness of 7 ± 1 layers (up to 2.8 nm) and an atomically flat hBN/Si interface, as evident from the cross-section high-resolution transmission electron microscopy (TEM) images in Figure 1(a) and 1(b). The thickness corresponds to a growth rate of ∼ 2 ML/h, slightly lower than what was observed on Ge(001)/Si substrates using similar growth conditions.^31^ This is compatible with the indication given by the results of ab initio calculations that the growth front of multilayer hBN may be different on these substrates and less reactive for hBN/Si(001), where zigzag (less reactive) edges seem to be allowed in contrast to predominantly armchair (more reactive) edges expected for hBN/Ge(001). More details can be found in the Supporting Information. The exact chemical species contributing to the growth of hBN from borazine are unknown, but they appear to be inert with respect to Si, as indicated by the absence of any buffer layer at the hBN/Si interface. In particular, decomposition of the borazine molecule down to atomic N and B can be ruled out.

**Figure 1:**
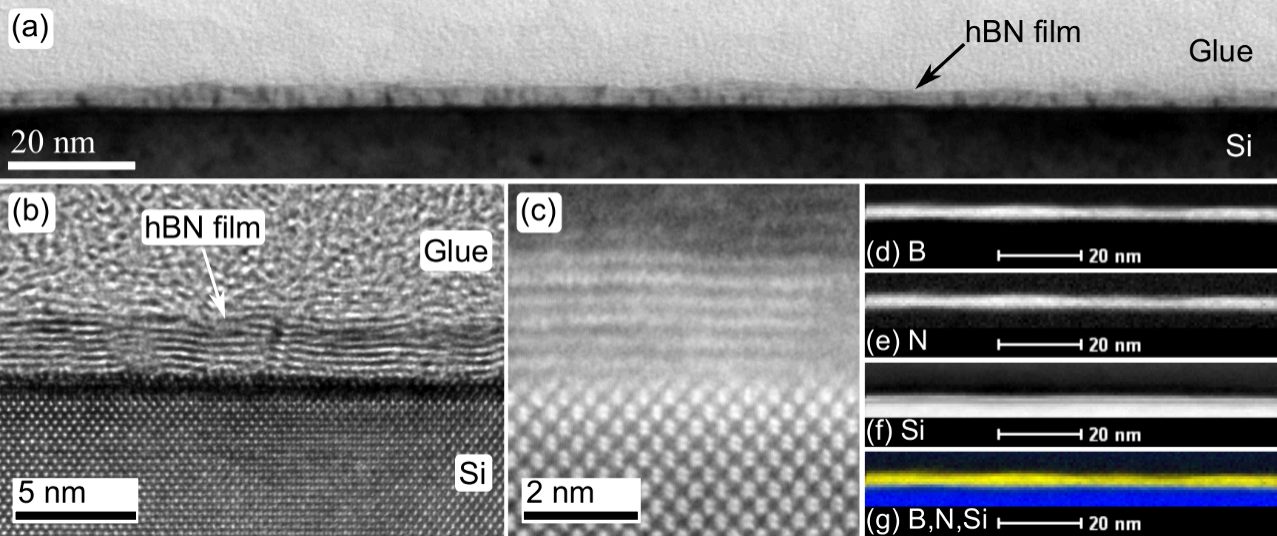
(a) Low-magnification and (b) high-resolution TEM image of an hBN/Si(001) film. (c) Atomic resolution MAADF image showing the crystallinity of Si at the hBN/Si interface. (d-f) EELS intensity maps of the B K-edge, N K-edge, and Si L-edge, respectively. (g) RGB overlay of EELS maps.

The bright-field image in Figure 1(b) shows a dark region directly below the hBN/Si interface, which is attributed to electron-optical effects during imaging, not physical changes to the Si crystal. In order to better characterize this interface region, atomic resolution scanning transmission electron microscopy (STEM) analyses were performed. Figure 1(c) presents a medium-angle annular dark-field (MAADF) image, which clearly shows that the Si substrate remains perfectly crystalline up to the first hBN layer and forms an atomically abrupt interface. Electron energy loss spectroscopy (EELS) maps, depicted in Figure 1(d)-(g), further corroborate the abruptness of the interface as no Si signal is found in the hBN regions, and vice versa.

Atomic resolution annular bright-field (ABF) STEM imaging enables the determination of the rotational alignment of the hBN layers relative to the Si substrate. In this imaging mode, columns of atoms appear as dark spots, as can be seen in Figure 2(a). In several places in the hBN film, clearly separated dark spots with an average distance of 2.17 Å are visible, indicating that they correspond to B–N pairs as viewed along the [011̅0] direction of hBN. Since the STEM image was taken along the [110] zone axis of Si, an orientational alignment of hBN [011̅0] || Si [110] can be concluded. This suggests that during growth, zigzag edges of hBN islands align themselves along a ⟨110⟩ direction on the Si(001) surface (see also insets of Figure 2(a)), as reported previously for hBN growth on Ge(001) substrates.^27,30^ While the hBN layers are generally well-oriented parallel to the Si interface (as opposed to forming randomly oriented nanocrystallites), they are visibly corrugated, which is the likely reason atomic resolution was not achieved everywhere in the image. The corrugation could be caused by grain boundaries, which form upon coalescence of (triangular) hBN islands that were aligned to two different of the four equivalent Si⟨110⟩ directions. Alternatively, a rotational misalignment of these layers would have the same effect. Note that the [213̅0] zone axis of hBN was not observed anywhere in the sample.

**Figure 2:**
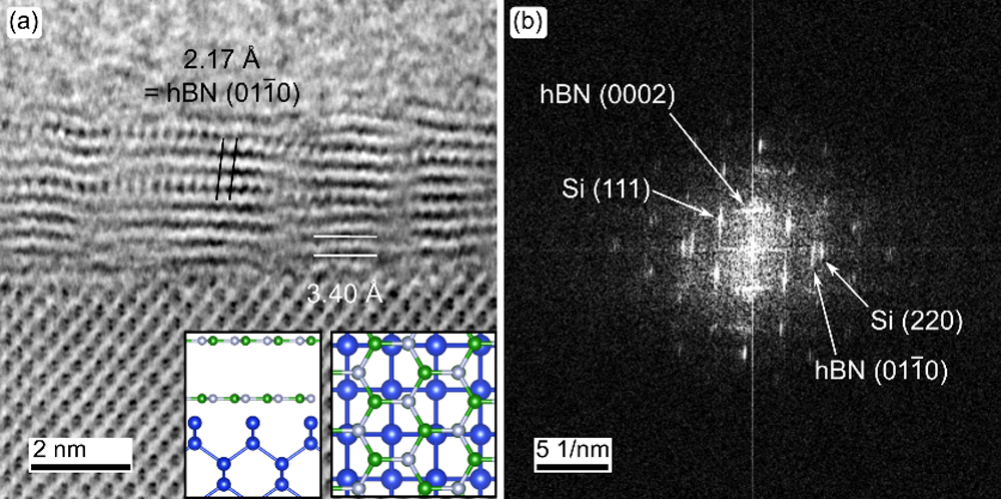
(a) Atomic resolution ABF-STEM image of an hBN/Si(001) film, showing that the hBN [011̅0] direction is aligned parallel to the Si [110] direction. Insets show the determined alignment of the hBN film and Si substrate, as viewed along the [110] direction (left) and [001] direction (right). (b) Corresponding diffraction pattern obtained from FFT.

The alignment of the hBN layers is corroborated by the diffraction pattern shown in Figure 2(b). In addition to the spots corresponding to the Si(220) and Si(111) planes, slightly diffuse spots corresponding to the corrugated hBN(0002) planes can be observed. On the same line as the Si(220) spots, another set of spots corresponding to the hBN(011̅0) planes is visible. The average interlayer distance was determined to be 3.40 Å, which agrees well with the expected value of 3.33 Å for hBN. Further details about the analysis of the atomic resolution STEM images and diffraction patterns can be found in the Supporting Information, including an additional EELS investigation.

Despite the corrugation of the layers, the hBN surface is relatively smooth, with an RMS roughness of 1.1 nm as measured by atomic force microscopy (AFM). Higher growth temperatures are expected to improve the smoothness of the layers, but were not possible with our setup. Noticeably, the surface is free of wrinkles, as can be seen in Figure 3(a), in contrast to what is often observed on sapphire substrates.^23–26^ This suggests a weak adhesion between the hBN film and Si substrate, allowing for the compensation of strain caused by the mismatched coefficients of thermal expansion. The larger-magnification AFM image in Figure 3(b) reveals the presence of small islands on the sample surface, with a diameter of 30–60 nm and a height of 3–6 nm. We assume these are the same boron-rich, amorphous BN islands formed in the early stages of growth that have recently been reported for hBN growth on Ge(001)/Si substrates using the same setup.^31^ In any case, they do not meaningfully contribute to the surface roughness: an RMS roughness of 1.0 nm is obtained if the islands are omitted from the calculation, a mere 9% reduction.

**Figure 3:**
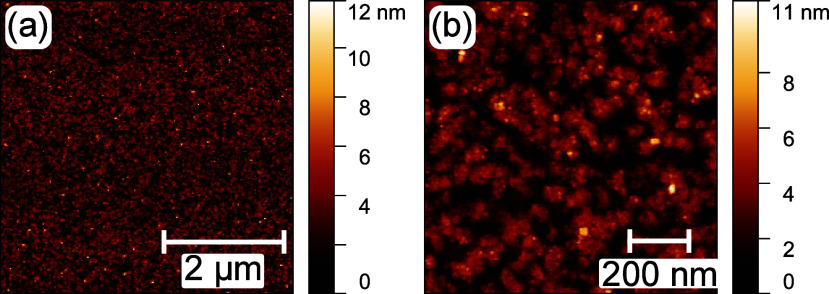
AFM images of an hBN film grown on Si(001), with a scan size of (a) 25 μm^2^ and (b) 1 μm^2^.

The crystalline quality of the grown hBN films was further investigated using Raman spectroscopy, after transfer of the films to 300 nm SiO_2_/Si substrates for interference enhancement. This is necessary because of the nonresonant nature of the Raman scattering in hBN,^37^ which means that the intensity of the E_2g_ peak of few-layer hBN films is too weak to be measured directly on the growth substrate. Based on the model proposed by Wang et al. for the case of graphene/SiO_2_/Si,^38^ an enhancement factor of ∼ 70 can be expected for hBN on a buffer layer of 300 nm SiO_2_. Figure 4(a) plots a typical Raman spectrum of our hBN films and Figures 4(b) and 4(c) show mappings of the hBN E_2g_ peak position and full width at half maximum (FWHM), respectively, acquired from a rectangular grid of 20 × 20 points with a distance of 1 μm. Both maps are quite homogeneous with few outliers, demonstrating the homogeneity of the hBN films. An average peak position of 1369.1 ± 1.0 cm^–1^ is calculated, which corresponds to a slight upward shift compared to bulk hBN and is attributed to the presence of a small amount of compressive strain, possibly as a result of the transfer process.^39,40^ The calculated average FWHM is 31.9 ± 1.3 cm^–1^, which means a lower limit of 6.1 nm lateral crystallite size can be estimated using the Nemanich model.^41^ The value is comparable to, if slightly higher than, those reported for 15–20 nm thick, well-oriented hBN films grown on sapphire substrates.^23–26^ However, our films were grown at significantly lower temperature (900 °C vs 1300–1500 °C), indicating that the temperature requirements to achieve high-quality hBN films might be lower on Si(001) compared to sapphire substrates when using borazine as the precursor.

**Figure 4:**
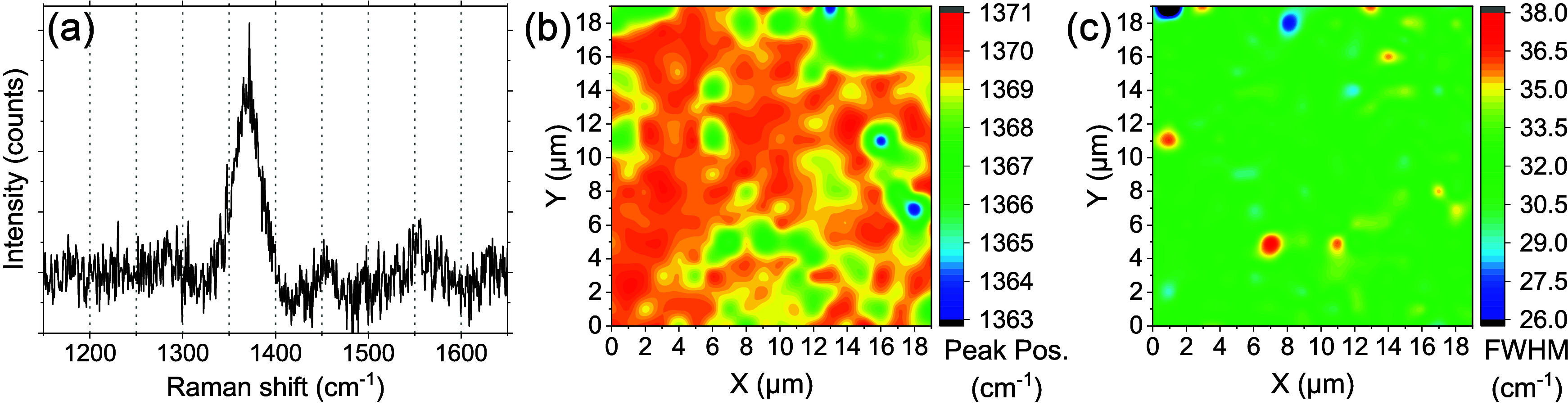
(a) Raman spectrum of an hBN film grown on Si(001), after transfer onto SiO_2_/Si. (b-c) Mappings of the hBN E_2g_ peak position and FWHM, respectively.

The stoichiometry of the deposited films is excellent, with a N:B ratio of 1.02 ± 0.1. A more detailed analysis of the N 1s and B 1s X-ray photoelectron spectra can be found in the Supporting Information, alongside some first DFT simulation results regarding the growth mechanism of hBN on Si(001) from borazine.

Finally, spectroscopic ellipsometry was explored as a fast, nondestructive method to determine the film thickness, refractive index, and optical bandgap (via the extinction coefficient) of the grown hBN films directly on the Si(001) substrate. The refractive index may serve as a figure of merit for the crystallinity, since amorphous BN has a much lower refractive index compared to hBN, with n­(aBN) = 1.4 and n­(hBN) = 2.2 at 633 nm,^42,43^ and thus complements the Raman FWHM, which mainly represents the crystallite size. The optical bandgap, on the other hand, depends on the presence of electronic states that extend into the bandgap of the grown material, caused e.g. by vacancies, interstitials, or substitutional impurity atoms such as carbon and oxygen.^44,45^ The refractive index of hBN has been measured over a wide wavelength range using both exfoliated flakes and CVD-grown films with thicknesses between 150 nm and 200 μm,^46–50,43^ and those results have been used to successfully differentiate between mono-, bi-, and trilayer hBN using ellipsometry.^51,52^ In contrast, we are interested in studying the optical functions of few-nm hBN films, including the extinction coefficient in the DUV range, as quality markers alongside the film thickness, as this requires no additional experimental effort.

Figure 5(a) plots the measured ellipsometric angles Ψ and Δ (points), as well as the model fit (solid lines), as a function of the photon energy in the DUV and visible spectral range. The optical model consists of three layers: air, hBN, and Si. The ambient air is assumed to have a constant refractive index of 1 and no absorption. For the Si substrate, a Leng oscillator model fitted to a blank Si reference spectrum is used, and the hBN film is described using a Tauc-Lorentz model with two oscillators. Note that while the refractive index of hBN is anisotropic, we only consider the ordinary (in-plane) component in our model. This is motivated by the fact that the extraordinary (out-of-plane) component can contribute only weakly to the signal in our ultrathin films.^53,54^ The lack of contribution is further exacerbated by the low angle of refraction in hBN due to the large refractive index of hBN compared to air, meaning that the light propagates almost perpendicularly to the surface and consequently, the electric field mainly interacts with the in-plane component.^55^ Indeed, using an anisotropic optical model did not improve the fit, and neither did adding more oscillators.

**Figure 5:**
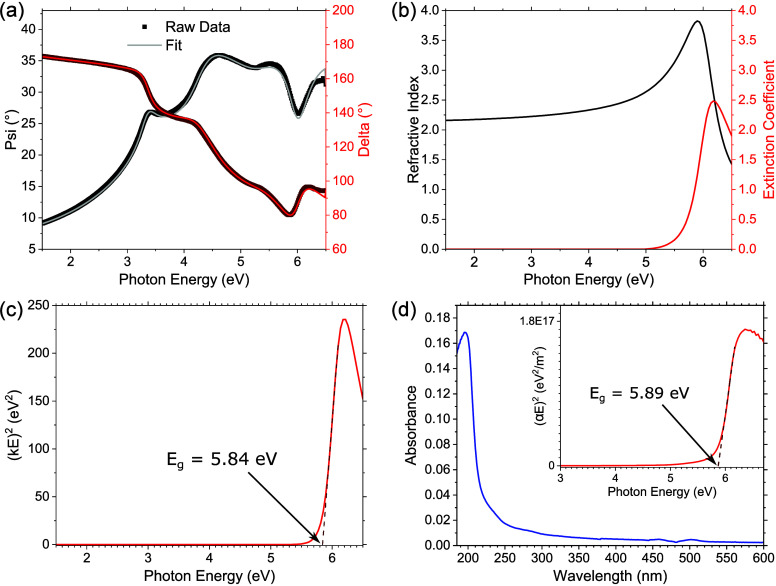
(a) Measured ellipsometry spectra and model fit of hBN/Si(001). (b) Fitted optical functions of the hBN layer. (c) Tauc plot constructed using the extinction coefficient to determine the optical bandgap of hBN. (d) Confirmation of optical bandgap via UV–vis absorption spectroscopy, inset shows Tauc plot.

The fitted model agrees well with the measured data and an hBN thickness of 2.40 ± 0.03 nm is obtained from a mapping of 11 × 11 points with a distance of 300 μm using a spot size of approximately 200 μm (see the Supporting Information for the map). This thickness corresponds to 7 ML of hBN, which is consistent with the TEM results. The optical functions resulting from the fitted model are plotted in Figure 5(b). The refractive index *n* is 2.17 at 1.96 eV (= 633 nm) and increases nonlinearly to 3.82 in the DUV range, in agreement with previous reports of the ordinary refractive index of hBN.^46–48,43^ This indicates a good crystallinity of the grown hBN films, which is supported by the TEM images. The extinction coefficient *k* is zero below 5 eV and exhibits a sharp absorption edge near 6 eV. An optical bandgap of 5.84 eV is obtained by plotting *(kE)* ^*2*^ vs *E* and extrapolating a fit of the linear region to the *x*-axis, as illustrated in Figure 5(c). This is equivalent to the Tauc plot method,^56^ where the absorption coefficient α is used in place of *k*, since both quantities are proportional to each other.

Furthermore, in order to verify the optical bandgap, UV–vis absorption spectroscopy was conducted on an hBN film transferred onto a quartz glass microscopy slide. Figure 5(d) plots the absorbance spectrum of the hBN film in the wavelength range of 185–600 nm and the inset shows the corresponding Tauc plot. It yields an optical bandgap of 5.89 eV, confirming the ellipsometry results. The value is close to the one expected for perfect hBN (∼ 6 eV), indicating a low density of point defects in the grown hBN films.

In conclusion, we demonstrated the synthesis of ultrathin hBN films on Si(001) substrates, achieved by using a continuous flow CVD process with borazine precursor at 10^–3^ mbar and 900 °C. The individual hBN layers are well-oriented parallel to the Si interface and the film exhibits good crystallinity relative to prior reports on hBN/Si. The single-source borazine precursor avoided the formation of an amorphous interlayer at the hBN/Si interface. The observed differences in the growth on Si(001) and Ge(001) surfaces is explained on the basis of ab initio simulations. Additionally, spectroscopic ellipsometry was explored as a fast and nondestructive method to obtain the refractive index and optical bandgap of the grown hBN films directly on the growth substrate. These film properties can serve as process control parameters complementing Raman and TEM investigations for future large-scale fabrication of hBN films.

## Supplementary Material



## Notes

Experimental methods, influence of key process parameters on the growth of hBN on Si(001), determination of interlayer distance and hBN orientation from atomic resolution STEM, EELS analysis, thickness mapping with spectroscopic ellipsometry, analysis of the N 1s and B 1s XPS spectra, and first DFT simulation results regarding the growth mechanisms of hBN on Si(001) vs Ge(001) from borazine (PDF)
