# Supplementary material for: Direct Synthesis of Ultrathin Hexagonal Boron Nitride Films on Si(001)
Source: Nano Lett. 2026 Jun 3;26(23):7701–7. doi: 10.1021/acs.nanolett.6c01435 (PMC13281532; doi:10.1021/acs.nanolett.6c01435)
Supplement: Supplementary file 1 [file nl6c01435_si_001.pdf]

Supporting Information

Direct Synthesis of Ultra-Thin Hexagonal Boron Nitride Films on Si(001)

Max Franck [a,*], Jaroslaw Dabrowski [a], Markus Andreas Schubert [a], Gianfranco Sfuncia [b], Giuseppe Nicotra [b], Christian Wenger [a,c], and Mindaugas Lukosius [a,*]

[a] IHP – Leibniz Institute for High Performance Microelectronics, Im Technologiepark 25, 15236 Frankfurt (Oder), Germany

[b] Istituto per la Microelettronica e Microsistemi (CNR-IMM), Ottava strada, 5 (Zona Industriale), 95121 Catania, Italy

[c] Semiconductor Materials, BTU Cottbus-Senftenberg, Platz der Deutschen Einheit 1, 03046 Cottbus, Germany

* Email: franck@ihp-microelectronics.com, lukosius@ihp-microelectronics.com

Experimental Methods

A RIBER Compact 21T ultra-high vacuum (UHV) CVD system was used to grow hBN films on Si(001) substrates. The Si(001) samples (39×9 mm²) were annealed in UHV (~10^{-8} mbar) for 30 min at a setpoint temperature of 900 °C, which removed the native oxide by converting it into volatile SiO. For hBN synthesis, the setpoint temperature was raised to 980 °C and 40 sccm of Ar carrier gas (99.9999 %, Air Liquide) were bubbled through the liquid borazine precursor ($B_3N_3H_6$, Katchem), which was held at -10 °C, resulting in a reactor pressure of 10^{-3} mbar. The Ar/borazine mixture is delivered into the reactor chamber via a stainless-steel injector tube, which terminates approximately 8 cm away from the sample surface. After a growth time of 240 min, the gas flow was stopped and the samples were rapidly cooled down to room temperature. Actual surface temperatures are estimated to be ~80 °C lower than the respective setpoint temperatures via calibration to the melting point of Ge.

The chemical composition of the grown hBN films was characterized by X-ray photoelectron spectroscopy (XPS, SPECS GmbH) using an Al Kα X-ray source. The crystalline structure was

assessed by high-resolution transmission electron microscopy (HRTEM), using a FEI Tecnai Osiris microscope operated at 200 kV, which offers a lateral resolution of 0.26 nm in HRTEM mode. The spatial distribution of boron and nitrogen was studied via electron energy loss spectroscopy (EELS) in the scanning TEM mode of the same instrument. Scanning transmission electron microscopy (STEM) was performed on a cold-FEG, probe Cs-corrected JEOL ARM-200F operated at 200 kV, providing a lateral resolution of 0.65 Å. Annular bright-field (ABF) images were acquired by collecting transmitted electrons within the 11–22 mrad angular range. Electron energy-loss spectroscopy (EELS) was performed using a Gatan GIF Quantum ER spectrometer operated in dual-EELS mode.

Atomic force microscopy (AFM, Bruker Dimension Icon) was used to investigate the morphology of the samples. The crystalline quality was characterized by Raman spectroscopy using a Renishaw inVia spectrometer (532 nm excitation wavelength, spot diameter ~1 µm). Raman spectra were referenced to the Si peak at 520.5 cm^{-1}. Spectroscopic ellipsometry (SENTECH SENresearch 4.0) was conducted in the 190 – 1040 nm wavelength range at an angle of incidence of 70° to determine the thickness, optical functions, and optical band gap of hBN. UV-vis absorption spectroscopy (PerkinElmer LAMBDA 1050+, 185 – 600 nm wavelength range, transmission mode) was used to verify the optical bandgap. UV-vis and Raman spectroscopy required transfer to quartz glass and 300 nm SiO_2/Si substrates, respectively. A standard PMMA-assisted, wet-chemical transfer process was used, similar to the one described in Ref. [1].

Ab initio calculations have been performed using the plane-wave pseudopotential density functional package Quantum Espresso.[2] [3] The numerical conditions and physical approximations were similar to those reported in Refs. [4] [5] [6] [7]. In particular, van der Waals forces were treated within the RVV10 scheme[8] and the most critical energy differences included the contribution from zero-point energy (ZPE) differences.

Influence of Key Process Parameters on the Growth of hBN on Si(001)

From earlier studies on Ge(001) substrates,[9] [4] the growth temperature, the local borazine (BZ) partial pressure, and the temperature of the precursor gas prior to entering the reactor chamber were identified as key parameters influencing the quality of the hBN films, which holds true for Si(001) substrates as well. Low growth temperatures (≤ 820 °C) result in mostly amorphous BN with small hBN crystallites (large Raman FWHM), while temperatures around 900 °C yield nanocrystalline films with Raman FWHMs slightly higher than those reported in the main text, and a random orientation of the crystallites. Initially, we observed exceedingly low growth rates, which led us to choose relatively high local borazine partial pressures at the sample surface by injecting the Ar/BZ mixture close to the sample. However, this always led to the aforementioned randomly-oriented crystallites. At 900 °C, decreasing the local borazine partial pressure by moving the injector tube further away from the sample (which also reduces the temperature of the precursor gas prior to entering the reactor chamber, limiting unwanted gas-phase reactions), then yields the desired well-oriented hBN layers.

The positive influence of higher growth temperatures on the Raman FWHM is shown in Fig. S1 for 820 °C and 900 °C. It is likely that increasing the growth temperature beyond 900 °C would further improve the quality of the hBN/Si films, but this was not possible with our setup.

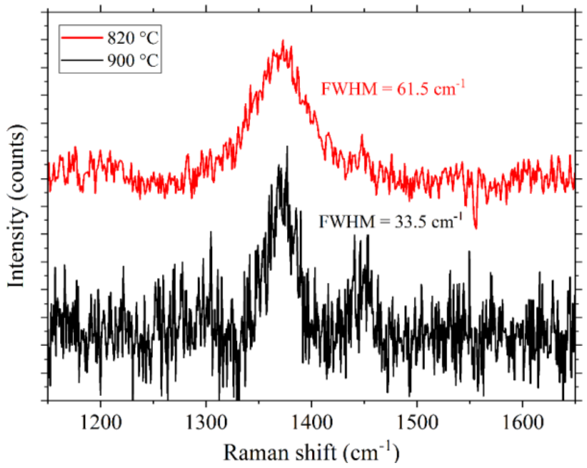

Figure S1: Raman spectra and FWHMs of two hBN films grown on Si(001) at 820 °C and 900 °C.

Figure S2 shows a TEM image of an hBN film grown on Si(001) at 900 °C, but with the borazine injector too close to the sample (local borazine partial pressure too high, possibly gas-phase reactions in the injector). The structure consists of randomly oriented nano-crystallites, and the surface is very rough.

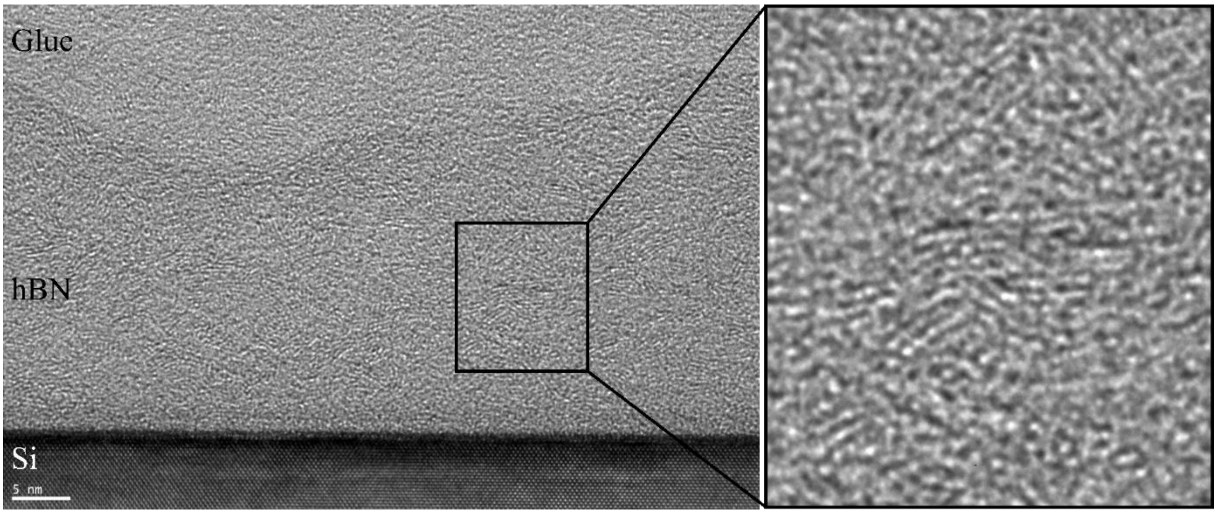

Figure S2: Cross-section TEM image of an hBN film grown on Si(001) with optimized temperature, but non-optimized local borazine partial pressure.

With the progression towards hBN films with layers that are well-oriented parallel to the Si interface, the surface roughness drastically reduces, as shown in the AFM images (Fig. S3).

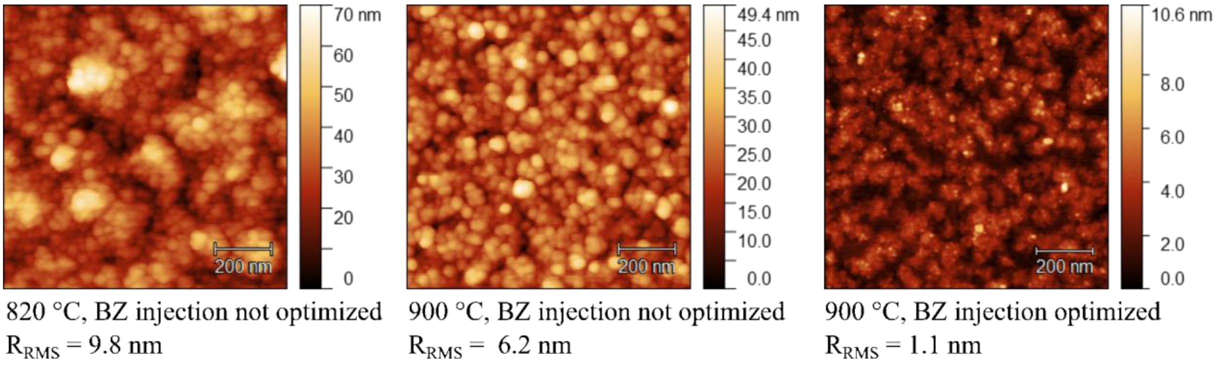

Figure S3: AFM images of hBN films grown on Si(001) substrates under different process conditions.

In addition to the decreased growth rate, we hypothesize that injecting the Ar/BZ mixture further away from the heated sample aids the formation of well-oriented hBN layers by limiting

pre-reactions of the borazine molecule before arriving at the sample surface. Computational fluid dynamics simulations showed that the optimized reactor configuration reduces the temperature of the precursor gas by ~200 °C in the high-pressure domain of the injector.[4] Elevated temperatures might cause pyrolytic dehydrogenation and dimerization reactions of the borazine molecule, and the resulting chemical species might be more reactive than borazine itself, leading to sub-optimal hBN growth by rapidly attaching themselves to the film in random orientations.

Determination of Interlayer Distance from Atomic Resolution STEM

The hBN interlayer distance was determined using both the diffraction pattern corresponding to the ABF image shown in Fig. S4(a) and the intensity variation in the ABF image shown in Fig. S4(c). In the first case, the radial distribution function of the diffraction pattern, shown in Fig. S4(b), was calculated. It shows two peaks at 2.91 nm^{-1} (d_{hkl} = 3.44 Å) and 3.15 nm^{-1} (d_{hkl} = 3.18 Å), corresponding to hBN(0002) planes and Si(111) planes, respectively. The inset of Fig. S1(b) shows the diffraction pattern and a red circle with a radius of 2.91 nm^{-1}, which crosses through the middle of the diffuse diffraction spots corresponding to the corrugated hBN layers. In the second case, the intensity profile shown in Fig. S4(d) was obtained from the region marked by the cyan rectangle in Fig. S4(c). The intensity profile is obtained by integrating the intensity along the short side of that rectangle for each pixel in the direction of the long side. The valleys corresponding to the hBN layers can clearly be distinguished, and an average interlayer distance of 3.40 Å is calculated.

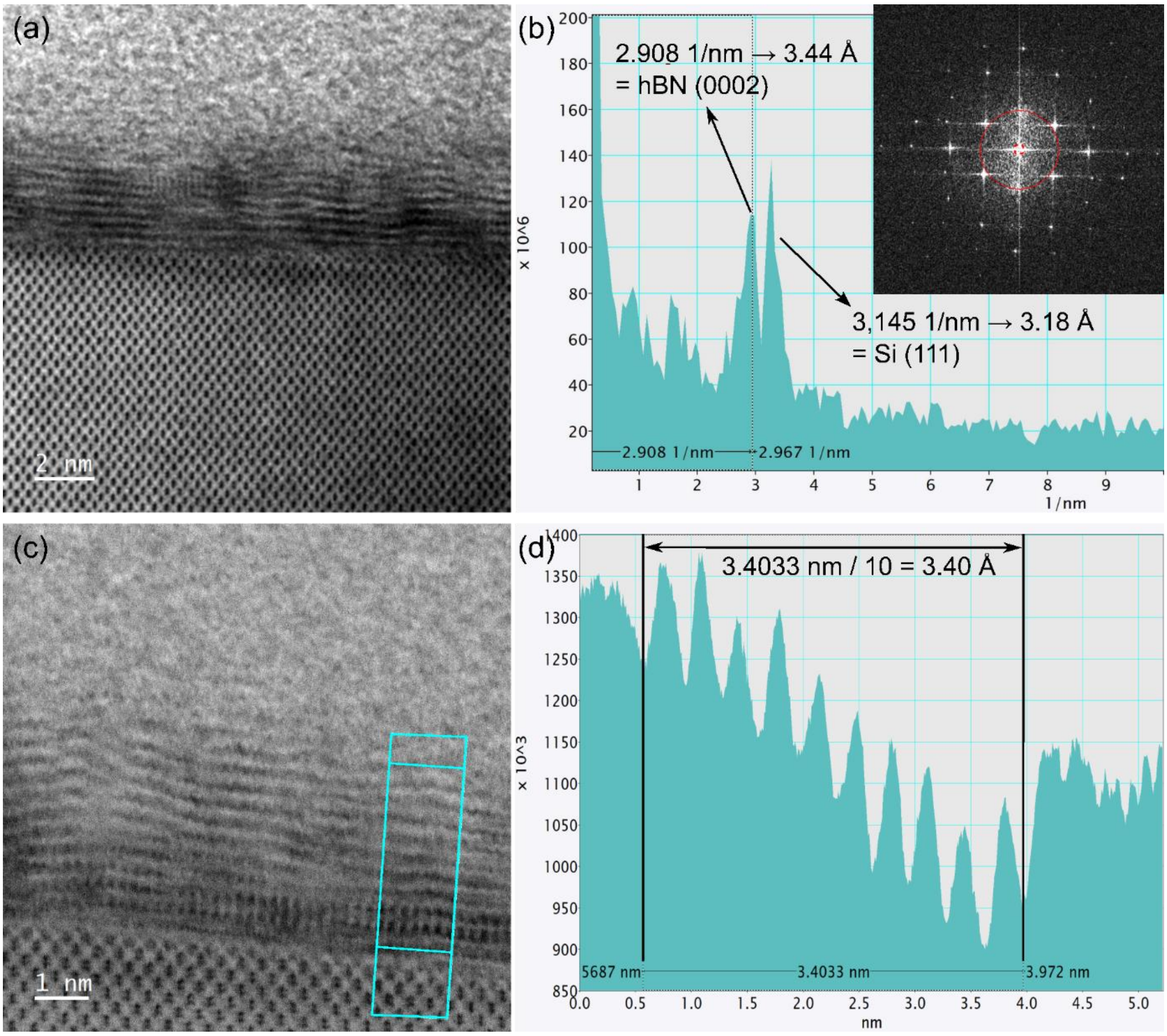

Figure S4: (a, c) ABF-STEM images of an hBN/Si(001) film. (b) Radial distribution function of the diffraction pattern shown in the inset (obtained via FFT from (a)), showing two peaks corresponding to the hBN(0002) and Si(111) diffraction spots. (d) Line profile parallel to the long side of the cyan rectangle in (c), obtained by integrating the intensity along the short side.

Determination of hBN Orientation from Atomic Resolution STEM

The orientation of the hBN layers relative to the Si(001) substrate has been determined from the ABF-STEM image shown in Fig. S5(a). An intensity profile was obtained from the region marked by the cyan rectangle, in the same fashion as described above, which is shown in Fig. S5(b). An average distance between the dark spots of 2.17 Å was determined, which corresponds to the distance between "pairs" of B and N atoms, if hBN is viewed along its $[01\bar{1}0]$ zone axis. Figure S5(c) illustrates the two possible zone axes of hBN, showing that the distance between the atomic features clearly distinguishes them. The distance between the B and N atoms of a "pair" in the case of the $[01\bar{1}0]$ zone axis is too small to be resolved by the STEM instrument.

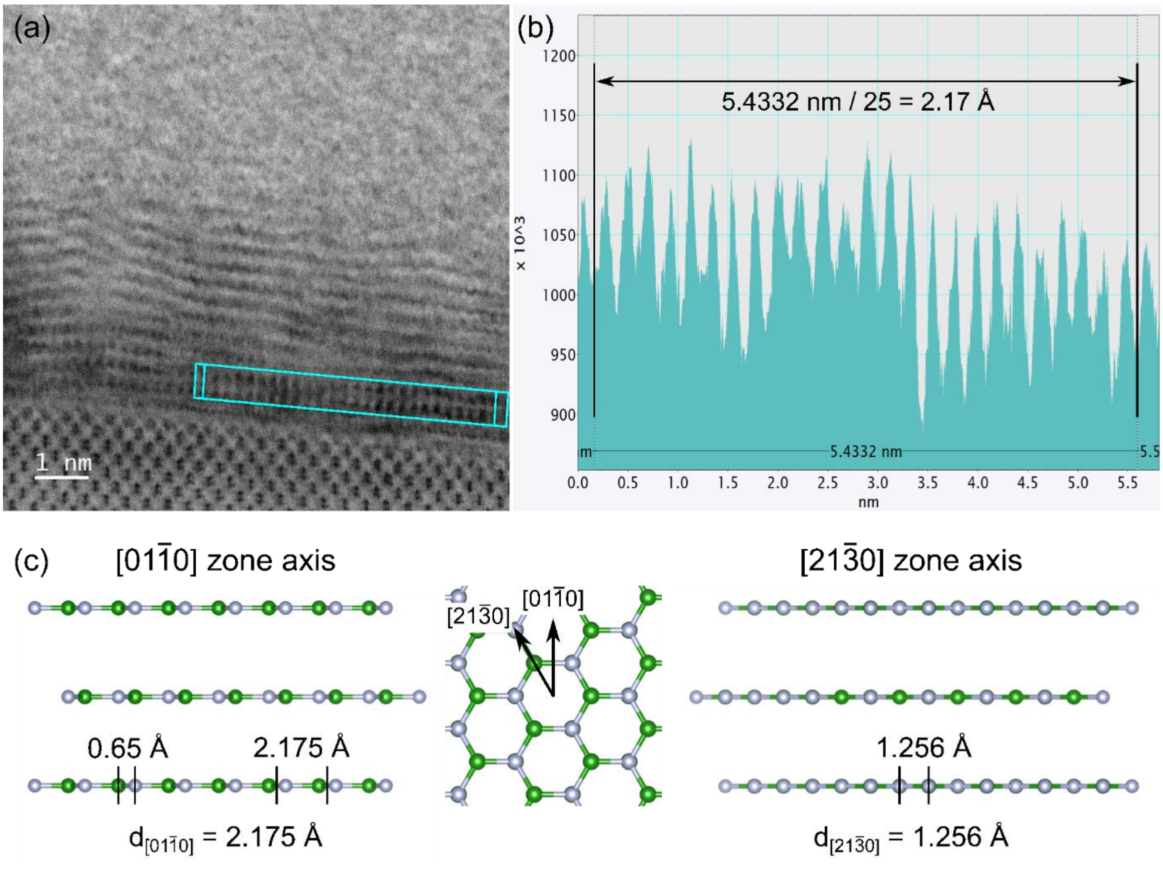

Figure S5: (a) ABF-STEM image of an hBN/Si(001) film. (b) Line profile parallel to the long side of the cyan rectangle in (a), obtained by integrating the intensity along the short side, giving the average spacing of the dark spots inside the rectangle. (c) The two possible zone axes of hBN, with expected distances between atomic features.

EELS Analysis

Figure S6 shows an atomic resolution annular dark field image of an hBN/Si(001) film, with three highlighted regions in the Si bulk, the Si surface, and the hBN film, as well as the corresponding EELS spectra. The Si bulk region (blue) only shows the Si L-edge, as expected, plus some carbon contamination from the preparation process of the FIB lamella. At the Si surface, a very small contribution of the O K-edge is visible, indicating the presence of a small amount of native oxide. Whether this is due to imperfect oxide removal prior to the hBN growth or due to later oxidation cannot be determined. The EELS spectrum in the hBN film region shows contributions from the B and N K-edges, but no O or Si.

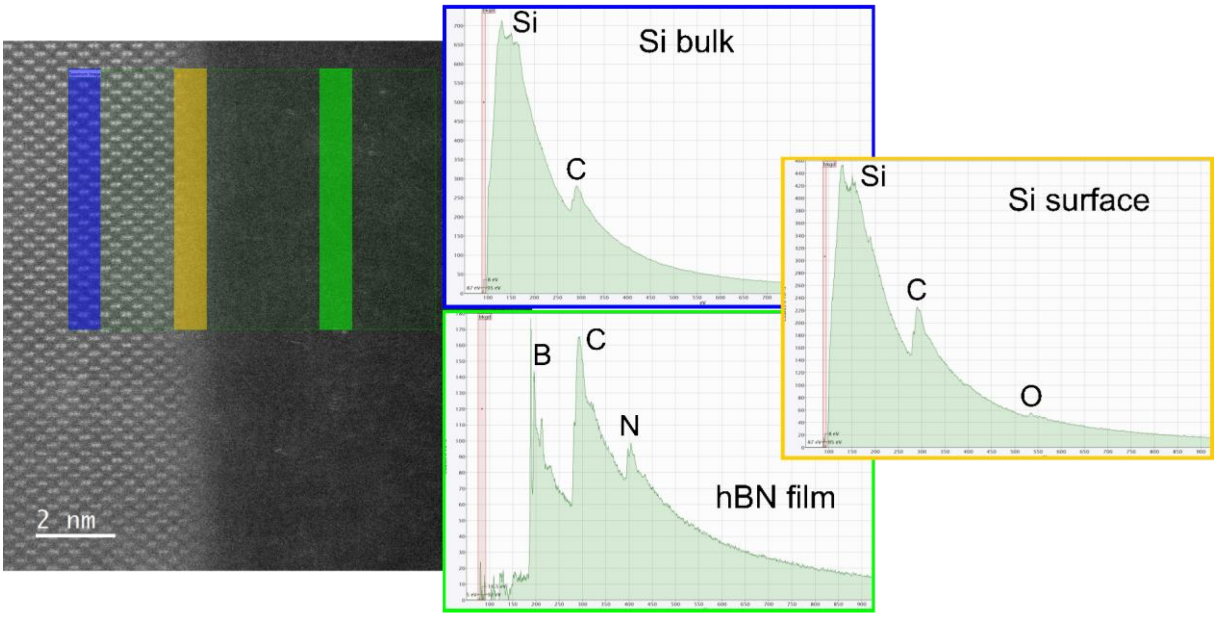

Figure S6: Annular dark-field image of an hBN/Si(001) film with EELS spectra corresponding to the highlighted regions (Si bulk, Si surface, and hBN film).

Thickness Mapping with Spectroscopic Ellipsometry

Figure S7 shows the mapping of the hBN thickness obtained by using micro spot lenses that focus the incident beam to a diameter of 200 μm (elongated to ~585 μm in the direction parallel to the plane of incidence), scanning a grid of 11×11 points with a distance of 300 μm, and fitting the model described in the main text at each point. The thickness is very homogeneous (2.40 ± 0.03 nm), with only a few outliers. Even including those, the minimum and maximum measured values differ by only 0.3 nm, or 1 ML.

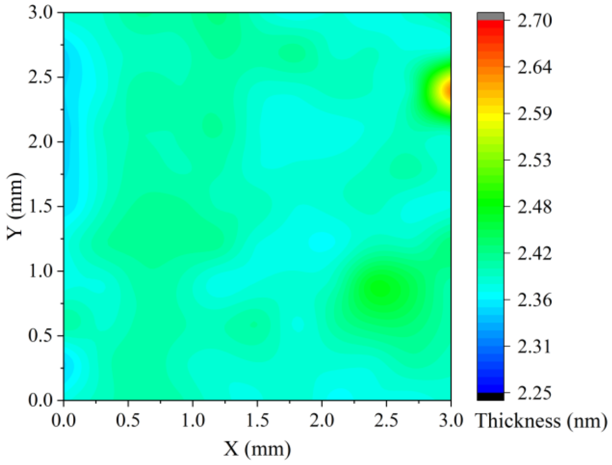

Figure S7: Mapping of hBN thickness by ellipsometry

XPS Analysis

The N 1s and B 1s XPS spectra of an hBN film grown on Si(001) using the optimized growth conditions are plotted in Fig. S8(a) and (b), respectively. The overall N:B ratio obtained from these spectra is 1.02 ± 0.1. In addition to the expected N–B and B–N peaks observed at 398.5 eV and 190.8 eV, respectively, peak deconvolution using a Shirley background and Gaussian-Lorentzian line shapes reveals the presence of a minor peak component in the N 1s spectrum, centered at 399.6 eV. This is attributed to N–H bonds,[10] which could indicate incomplete dehydrogenation reactions and might further point towards higher growth temperatures potentially benefitting the grain size of the hBN films, in addition to the TEM and Raman data presented in the main text. On the other hand, the presence of B–H bonds (~188 eV) would also be expected in this case. This discrepancy prompted an investigation into the possibility of hBN growth involving N-terminated zigzag edges on Si(001). This possibility would be in contrast to Ge(001) substrates, where growth is expected to proceed from armchair edges and neither N–H nor B–H bonds were observed under the same growth conditions.[4 6]

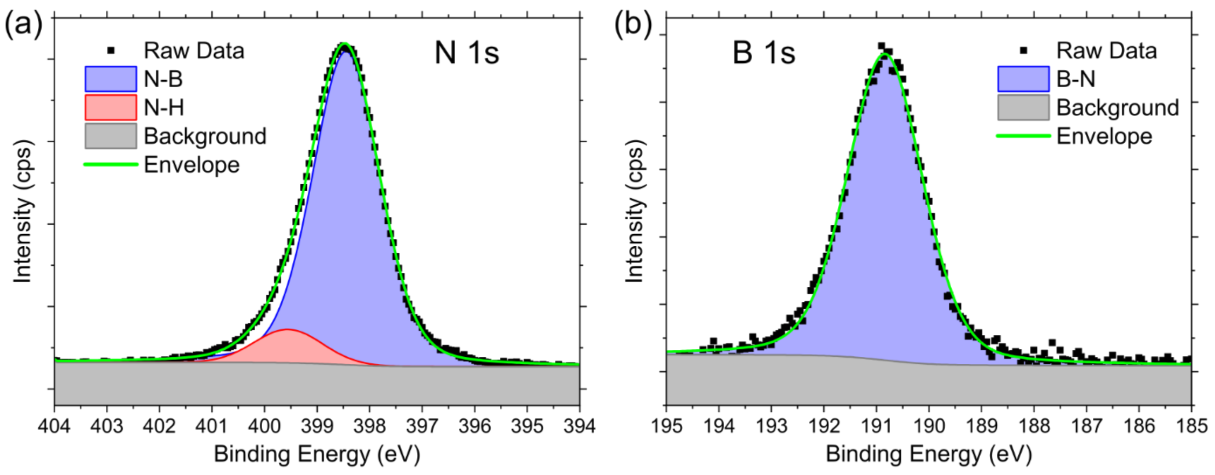

Figure S8: (a) N 1s and (b) B 1s XPS spectra of an hBN film grown on Si(001) under optimized growth conditions, with fitted peak components.

DFT Results

First ab initio simulations point towards a significant difference in the growth of the first hBN monolayer on Ge(001) and on Si(001): while on Ge(001) the most probable product of borazine decomposition is $B_3N_3H_5$(ads) + H(ads), on Si(001) it is BN_2H_3(ads) + B_2NH_3(ads), see Fig. S9(a), whereby splitting into moieties with fewer B or N atoms appears to be kinetically difficult on both surfaces. Therefore, similar deposition conditions are likely to promote the growth from complete hexagonal rings on Ge(001), leading to predominance of armchair edges. These edges tend to be free of hydrogen at the temperatures and partial pressures of H_2 and $B_3N_3H_6$ present during the CVD process. In contrast, partially dissociated (halved) precursor molecules may be available on Si(001), possibly leading to abundance of NH-terminated zigzag edges. In both cases the growth rate is limited by H_2 detachment from the growth front, but the growth from zigzag edges may proceed slightly slower due to more complex reaction pathways that involve dissociation of some BN bonds in the attached borazine molecule, see Fig. S9(b). Detailed analysis of these intricate processes is beyond the scope of the current report and will be given in a separate publication.

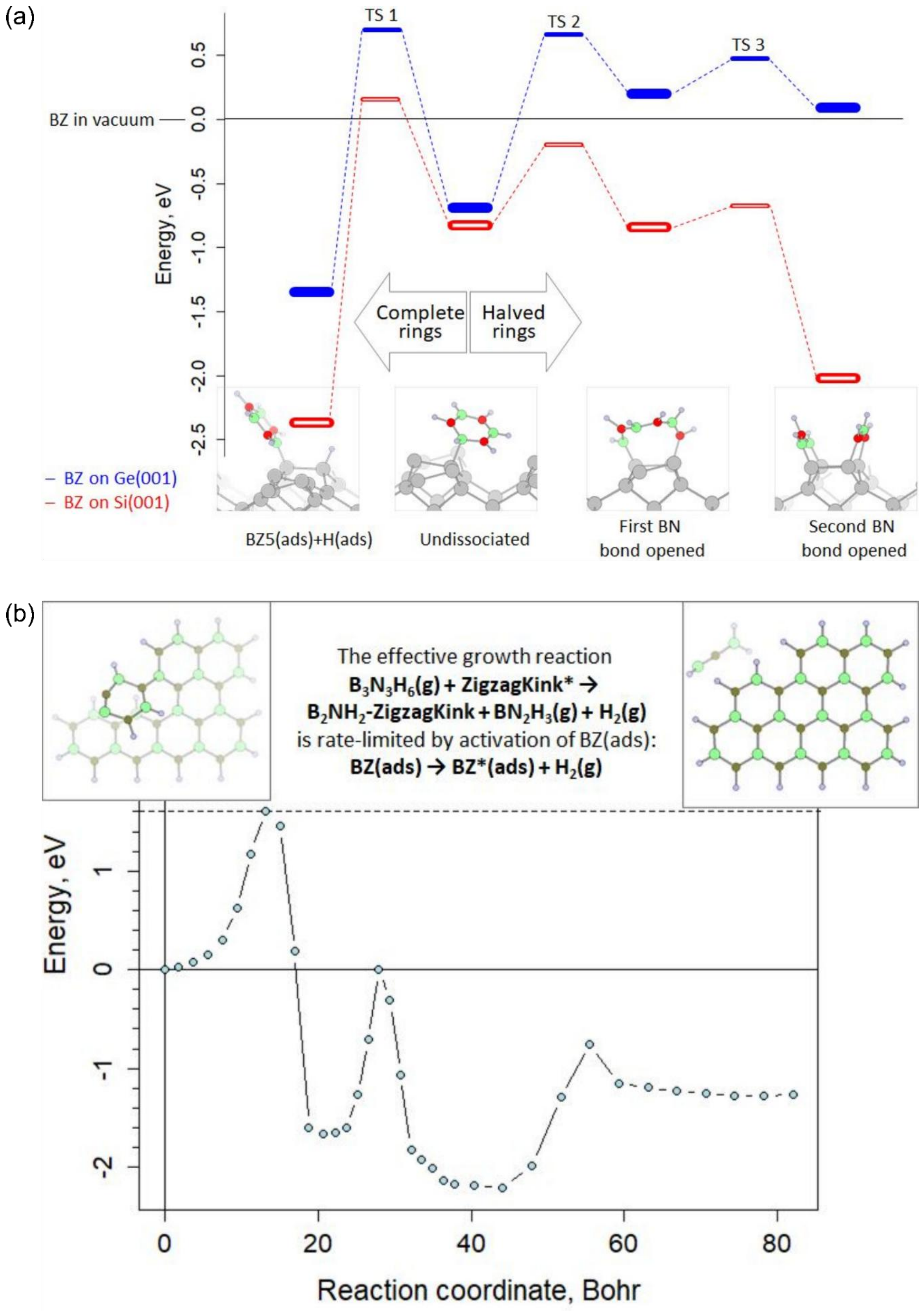

Figure S9: (a) Dissociative adsorption of borazine on Ge(001) and Si(001). (b) Dissociation of borazine on a kink of a zigzag hBN edge.

References

(1) Behura, S.; Nguyen, P.; Debbarma, R.; Che, S.; Seacrist, M. R.; Berry, V. Chemical Interaction-Guided, Metal-Free Growth of Large-Area Hexagonal Boron Nitride on Silicon-Based Substrates. *ACS Nano* **2017**, *11* (5), 4985–4994. DOI: 10.1021/acsnano.7b01666.

(2) Giannozzi, P.; Baroni, S.; Bonini, N.; Calandra, M.; Car, R.; Cavazzoni, C.; Ceresoli, D.; Chiarotti, G. L.; Cococcioni, M.; Dabo, I.; Dal Corso, A.; Gironcoli, S. de; Fabris, S.; Fratesi, G.; Gebauer, R.; Gerstmann, U.; Gougoussis, C.; Kokalj, A.; Lazzeri, M.; Martin-Samos, L.; Marzari, N.; Mauri, F.; Mazzarello, R.; Paolini, S.; Pasquarello, A.; Paulatto, L.; Sbraccia, C.; Scandolo, S.; Sclauzero, G.; Seitsonen, A. P.; Smogunov, A.; Umari, P.; Wentzcovitch, R. M. QUANTUM ESPRESSO: a modular and open-source software project for quantum simulations of materials. *Journal of physics. Condensed matter : an Institute of Physics journal* **2009**, *21* (39), 395502. DOI: 10.1088/0953-8984/21/39/395502. Published Online: Sep. 1, 2009.

(3) Giannozzi, P.; Andreussi, O.; Brumme, T.; Bunau, O.; Buongiorno Nardelli, M.; Calandra, M.; Car, R.; Cavazzoni, C.; Ceresoli, D.; Cococcioni, M.; Colonna, N.; Carnimeo, I.; Dal Corso, A.; Gironcoli, S. de; Delugas, P.; DiStasio, R. A.; Ferretti, A.; Floris, A.; Fratesi, G.; Fugallo, G.; Gebauer, R.; Gerstmann, U.; Giustino, F.; Gorni, T.; Jia, J.; Kawamura, M.; Ko, H.-Y.; Kokalj, A.; Küçükbenli, E.; Lazzeri, M.; Marsili, M.; Marzari, N.; Mauri, F.; Nguyen, N. L.; Nguyen, H.-V.; Otero-de-la-Roza, A.; Paulatto, L.; Poncé, S.; Rocca, D.; Sabatini, R.; Santra, B.; Schlipf, M.; Seitsonen, A. P.; Smogunov, A.; Timrov, I.; Thonhauser, T.; Umari, P.; Vast, N.; Wu, X.; Baroni, S. Advanced capabilities for materials modelling with Quantum ESPRESSO. *Journal of physics. Condensed matter : an Institute of Physics journal* **2017**, *29* (46), 465901. DOI: 10.1088/1361-648X/aa8f79. Published Online: Oct. 24, 2017.

(4) Franck, M.; Dabrowski, J.; Schubert, M. A.; Vignaud, D.; Achehboune, M.; Colomer, J.-F.; Henrard, L.; Wenger, C.; Lukosius, M. Investigating Impacts of Local Pressure and Temperature on CVD Growth of Hexagonal Boron Nitride on Ge(001)/Si. *Adv. Mater. Interfaces* **2025**, *12* (1). DOI: 10.1002/admi.202400467.

(5) Dąbrowski, J. Ge(110) c(8×10) reconstructions stabilized by vibrations. *Surface Science* **2025**, *759*, 122761. DOI: 10.1016/j.susc.2025.122761.

(6) Dąbrowski, J.; Akhtar, F.; Franck, M.; Lukosius, M.; Reichmann, F. *IHPms21: Ab Initio Simulation of Materials for Si-Compatible Microelectronics*. https://www.gauss-centre.eu/results/materials-science-and-chemistry/ab-initio-simulation-of-materials-for-si-compatible-microelectronics.

(7) Dąbrowski, J.; Akhtar, F.; Franck, M.; Lukosius, M. Ab Initio Simulation of Materials for Environmentally Friendly Technologies. In *NIC Symposium 2025 Proceedings*; Peter, C., Müller, M., Trautmann, A., Eds.; Forschungzentrum Jülich, 2025; pp 193–203.

(8) Sabatini, R.; Gorni, T.; Gironcoli, S. de. Nonlocal van der Waals density functional made simple and efficient. *Phys. Rev. B* **2013**, *87* (4). DOI: 10.1103/PhysRevB.87.041108.

(9) Franck, M.; Dabrowski, J.; Schubert, M. A.; Wenger, C.; Lukosius, M. Towards the Growth of Hexagonal Boron Nitride on Ge(001)/Si Substrates by Chemical Vapor Deposition. *Nanomaterials* **2022**, *12* (19), 3260. DOI: 10.3390/nano12193260. Published Online: Sep. 20, 2022.

(10) Chastain, J.; King, R. C., Eds. *Handbook of X-ray Photoelectron Spectroscopy:A Reference Book of Standard Spectra for Identification and Interpretation of XPS Data*; Physical Electronics, 1995.
